# Comparison of reproductive history gathered by interview and by vital records linkage after 40 years of follow-up: Bogalusa Babies

**DOI:** 10.1186/s12874-019-0758-0

**Published:** 2019-06-04

**Authors:** Emily W. Harville, Marni Jacobs, Tian Shu, Dorothy Breckner, Maeve Wallace

**Affiliations:** 1 Department of Epidemiology, Tulane School of Public Health and Tropical Medicine, Epidemiology #8318, 1440 Canal ST STE 2000, New Orleans, LA 70112 USA; 2 Division of Biostatistics and Study Methodology, Children’s National Health System, Washington, DC 20010 USA; 3 Department of Global Community Health and Behavior, Tulane School of Public Health and Tropical Medicine, New Orleans, LA USA

**Keywords:** Common data elements, Vital statistics, Reproductive history, Cardiovascular disease, Bias

## Abstract

**Background:**

To examine the consistency and likely degree of bias in a study of cardiovascular health, linked with reproductive data over 40 years.

**Methods:**

Linkage of vital statistics data of births to female Bogalusa Heart Study participants was compared to interviewing of female participants. The characteristics of participants, the agreement, and demographic, study-related, and medical predictors of discrepancy were analyzed, using kappa statistics, mean and median differences, and logistic regression.

**Results:**

Overall, 3944 (66.7%) of participants were located by one or both sources. The strongest predictor of either linkage or interview was recent and/or frequent participation in the parent study. Agreement between the two sources was generally good (kappa > 0.9 for birthweight and 0.8 for gestational age). Black race, older age, and time since pregnancy were associated with greater discrepancy in reporting of outcomes, but cardiovascular risk factors generally were not.

**Conclusions:**

Combining information from multiple sources to increase sample size and outcome ascertainment may be valid, which will increase population health sciences’ ability to leverage the many existing, large-scale sources to answer previously unexplored questions, even those that the data were not initially collected to answer.

**Electronic supplementary material:**

The online version of this article (10.1186/s12874-019-0758-0) contains supplementary material, which is available to authorized users.

## Background

With the growing emphasis on use of existing data and cohorts [1, 2], as well as data harmonization to create large analyses across disparate datasets [3–5], it becomes more important to understand the degree to which these study designs provide accurate, reliable, and consistent data. While linkage of existing datasets and databases can be powerful and cost-effective, it can also magnify errors [6]. If multiple recordings of data fundamentally derive from the same source, or if linking tends to bias systematically the group of participants that are included in large-scale analyses, such study designs run the risk of leading to a greater degree of confidence in fundamentally flawed or biased analyses.

For example, migration limits the possibility of linkage between datasets. Most data are stored as part of a study database or as clinical or administrative data, and are limited by jurisdiction. Thus, any factor that affects the likelihood of migration affects the probability of linkage across databases. Socioeconomic status (SES) is likely to be particularly important, as it affects mobility, health, and quality of reporting, and can lead to serious bias in the conclusions of studies based on these datasets. Previous studies of mortality linkages have found reduced linkage with Hispanic populations, for instance [7, 8].

In addition to the general issue of the quality and value of data linkages, a question that has recently become more prominent is that of the relationship between reproductive history and health during other parts of the life course. There is a growing recognition that pregnancy does not operate independently of health during other periods of life [9–13]. While it has long been known that parity and age at pregnancy are risk factors for breast cancer [14], more recent research indicates a relationship between pregnancy complications and birth outcomes and later health, particularly cardiovascular disease and diabetes [15–17]. Studies of chronic disease, which are usually conducted in middle-aged or older populations, are therefore likely to be interested in finding data on the reproductive years. While several previous studies have looked at the comparison between self-report and other sources of data for studying pregnancy health [18–20], in most cases these were pregnancy or child cohorts, so the timing and usually location of the births was known precisely, and most often compared were medical records, rather than vital statistics data. In this analysis, we compare the results of a linkage with vital statistics data with women’s self-report of their pregnancy history in the context of a study designed to assess cardiovascular health, and in which the timing of pregnancies was not known and took place over a period of forty years.

## Methods

### Source cohort

The Bogalusa Heart Study (BHS) was begun in 1973 by Dr. Gerald Berenson [21]. Surveys of the town’s schoolchildren were repeated approximately every two years through 1994, examining newly enrolled children as well as re-examining those previously enrolled, with reexamination of adults begun in 1997 and continuing to the present day. Thus, BHS has examined the longitudinal history of childhood, adolescent, and now adult cardiovascular health. Risk factors measured have varied somewhat over the years, but consistently included anthropometrics, blood pressure, lipids, and glucose, with later extensions to echocardiography and arterial stiffness.

The Bogalusa Babies study was started in 2012. The goal of the study was to examine the relationship between preconception cardiovascular risk factors and reproductive histories within women in BHS. Three sources of information on birth outcomes were considered: vital statistics (birth certificates), interview, and medical records. All 5914 women who had ever participated in the BHS were eligible to participate in the Bogalusa Babies study, regardless of the number of previous study visits or whether the women had been pregnant. Participants were recruited through advertising, mailings, and systematic calls through the study database.

### Birth record data linkage

The data linkage has been described in detail previously [22]. Vital statistics birth record data were obtained from the three states thought most likely to include former BHS participants: Louisiana, Texas, and Mississippi. Briefly, Louisiana birth records were available from 1982 to 2009. Linkage of Louisiana birth record data to BHS data was completed using LinkPro v3.0 (InfoSoft, Inc., Winnipeg, MB) [23–25]. For 1982–1989 records, linkage variables available were maternal last name, Soundex code for last name, race, and year of birth. From 1990 to 2009, a three-stage linkage process was used, including deterministic record linkage based on maternal social security number (SSN), and probabilistic linkage when SSN was unavailable. Procedures conducted by the Texas and Mississippi vital statistics departments were based on their internal procedures and policies. Texas and Mississippi conducted two-stage linkages for data from 1988 to 2012 using Link Plus 3.0 [26]. Results were then examined for duplicates. If a birth was duplicated or occurred within six months of a previous birth, it was removed from the dataset.

### Interview

During the interview, women were asked whether they had ever been pregnant, the outcome of each pregnancy, and complications. Women were encouraged to consult a baby book (a scrapbook with memories of the pregnancy and first year), if they had one. They were asked to report the birthweight of each baby and whether the baby was born early, late, or on time, and how early or late, in days or weeks. If a woman said her baby was on time, gestational age was imputed as 39.5 weeks.

### Analysis

The analysis aimed to examine birth outcomes as recorded in the birth certificates and the interviews, both in terms of what predicted the likelihood of inclusion in various sources, and how closely the sources agreed. For this analysis, we focus on number of pregnancies, birthweight (including low birthweight, < 2500 g), and gestational age (including preterm birth, < 37 weeks’ gestation). A future analysis will focus on pregnancy complications such as gestational diabetes and pre-eclampsia, as we have a fourth source of information (the original BHS), and medical records are more crucial for understanding the differences (94% of interview participants provided permission/HIPAA releases for medical records, but in most cases the records were destroyed as over 10 years old.)

First, the births reported in interviews and linked in the datasets were compared. A birth was considered a definite match if it occurred to the same woman on the same date in both sources, then examined the possible sources of discrepancy, including mistakes in dates and births that occurred outside the date and geographic range of the linkage. Both singleton and multiple births were included; to our knowledge, all sets of multiples (1.3%) in the dataset were born on the same day. Probable matches included: births that occurred in the same year with no other date information, births in the same year within one month; births on the same month and day but one year apart, or births less than one year and three days apart. (All of these were considered plausible mis-reporting or mis-recording of the same births.) Both types of matches were included in analysis of agreement.

Next, we examined the characteristics associated with being included in one or both sources. Women were categorized as interview and linkage; interview only, reported at least one birth; interview only, did not report having given birth; linkage only; or neither interviewed nor found in the linkage. Demographic, study-related (number and recency of visits), and cardiovascular risk factors were compared across these categories, using chi-square, ANOVA, and nonparametric tests. When differences were found, regression analysis was used to determine whether those differences were due solely to age and year of participation.

Third, we limited the dataset to those with information from both sources. We examined agreement between sources with respect to birthweight, and gestational age, as well as dichotomized outcomes (very low birthweight, < 1500 g; low birthweight (LBW), < 2500 g; early preterm birth, gestational age < 34 weeks; preterm birth (PTB), gestational age, < 37 weeks). Kappa statistics and mean and median differences were calculated, controlling for clustering within woman (extended kappa statistics [27] and generalized estimating equations).

Finally, we examined predictors of agreement between sources, again looking at demographic, study-related, cardiovascular, and reproductive predictors of agreement and disagreement. Matched pregnancies were examined, with discrepancy defined as not agreeing on whether a pregnancy was LBW or PTB. We also examined these as predictors of size of the discrepancy. Results were again examined controlling for clustering within woman.

The Institutional Review Boards (IRB) of Tulane University (IRB ID#256406), the State Department of Health and Hospitals of Louisiana (Louisiana Department of Health), and the Texas Department of State Health Services approved this protocol (Mississippi deferred to the Tulane IRB). The linkage was conducted under a waiver of consent, as it was deemed minimal risk and infeasible without the waiver.

## Results

**Fig. 1:**
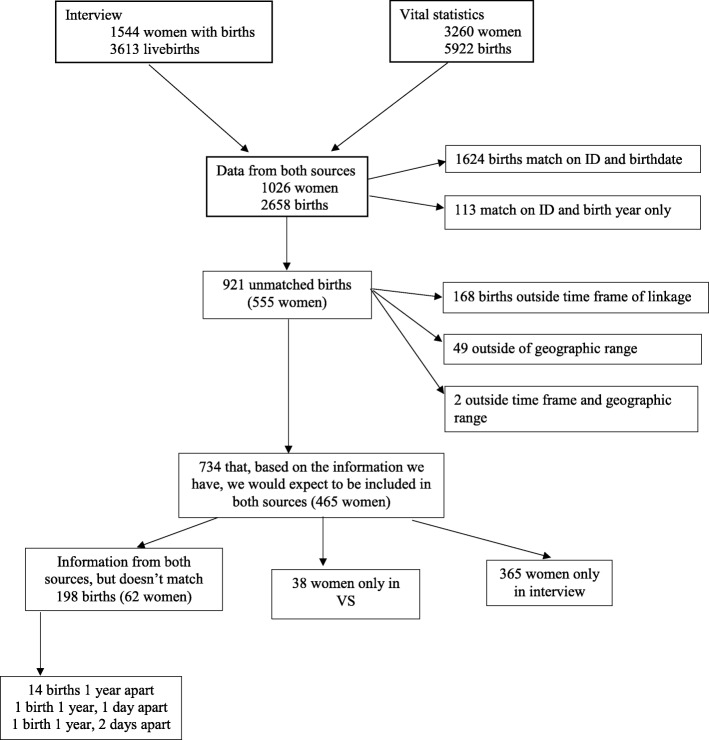
Flowchart, study population, Bogalusa Babies study

There were 1026 women with data from both vital records and interview, with a total of 2658 births reported (Fig. 1). Of these, 1624 were exact matches. An additional 113 matched on year only. 32 of these had year only provided from vital statistics due to confidentiality restrictions (Texas). Of the remaining 81, the median difference in time between the birth certificate and interview data was 2.0 days, with a mode of 10 days, a minimum of − 300 days, and a maximum of 228 days (date from birth certificate – date from interview).

Of the remaining 958 births reported in the interview, 65 occurred prior to 1982 and 105 after 2010, and 51 births were reported to occur outside of Louisiana, Mississippi, and Texas, and thus would not have been eligible to be linked in the linkage. 734 births to 465 women (62 women with non-matching information in both sources, 38 only vital statistics data, and 365 only interview data) were not included in both sources, but had no obvious reason for a lack of match in the other. Of these, 16 births were exactly one year or one year and 1–2 days apart.

Overall, 3944 (66.7%) of participants were located by one or both sources. The strongest predictor of either linkage or interview was recent and/or frequent participation in the parent study (Table 1). Those who were interviewed had more study visits (median 5) than those who did not (median 2, *p* < 0.01), and were more likely to have participated in the study as an adult. The groups that were interviewed were also more likely to have ever smoked, even after the age distribution and years of the interviews were controlled for (aOR for smoking for those with interview and linkage, 1.32, 1.05–1.66; with interview only 1.45, 1.13–1.86). Parental education was more likely to be missing for those who were not located (this data was not collected at early visits); among those with data, those who were located were more likely to have higher parental education. Differences in BMI, cholesterol, and blood pressure were largely explained by the age distribution of participation in the groups, although mean childhood BMI was higher in those who only interviewed (absolute values provided in table; adjusted beta for difference = 0.80, *p* < 0.01).

**Table 1:** Comparison of interviews, linkage, and overall dataset

	interview livebirth and linkage (*n* = 1024)		interview only, reported at least one livebirth (*n* = 458)		interview only, reported no births (*n* = 255)		linkage only (*n* = 2207)		neither linkage nor interview (*n* = 1970)		p for difference
	N	%	N	%	N	%	N	%	N	%	
number of visits											< 0.01
1	130	12.7	72	15.7	47	18.4	817	37.0	989	50.2	
2	136	13.3	23	5.0	23	9.0	515	23.3	371	18.8	
3–4	256	25.0	114	8.6	71	27.8	537	24.3	346	17.6	
5–6	195	19.0	89	12.6	44	17.3	216	9.8	160	8.1	
7 or more	307	30.0	160	34.9	70	27.5	122	5.5	103	5.2	
race											< 0.01
black	433	42.3	165	36.0	109	42.8	768	34.8	668	33.9	
white	591	57.7	293	64.0	146	57.3	1439	65.2	1302	66.1	
ever smoker											< 0.01
yes	368	42.3	206	49.3	80	41.5	490	30.8	436	33.5	
no	502	57.7	212	50.7	124	58.5	1103	69.2	866	66.5	
highest parental education											< 0.01
> high school	347	52.7	139	50.0	72	47.7	312	43.6	162	42.5	
high school or less	311	47.3	139	50.0	79	52.3	404	56.4	219	57.5	
mean SD											
age at youngest visit	8.8	3.8	12.9	9.8	9.6	5.5	8.8	3.5	10.0	5.1	< 0.01
age at oldest visit	25.9	12.6	31.1	13.7	25.3	13.3	15.4	8.1	15.1	9.2	< 0.01
age in 2018	44.6	7.3	50.5	8.3	45.2	8.5	42.5	7.4	44.3	9.3	< 0.01
median IQR											
year at first visit	1978	1974–1983	1974	1974–1979	1977	1974–1984	1981	1976–1988	1979	1974–1988	< 0.01
year at most recent visit	2000	1993–2014	2000	1993–2014	1999	1992–2014	1988	1985–1994	1988	1980–1994	< 0.01
mean SD											
BMI	21.6	5.2	22.5	5.4	22.7	6.4	19.2	4.2	19.5	4.8	< 0.01
child BMI	17.4	3.2	17.3	3.1	18.5	4.0	17.1	2.9	17.3	3.3	< 0.01
adolescent BMI	21.6	4.5	20.7	3.6	23.4	6.3	21.4	4.5	21.4	4.7	0.39
adult BMI	27.3	7.1	26.4	6.3	28.6	9.8	26.1	7.1	25.5	6.6	< 0.01
cholesterol	171.3	26.0	172.4	29.8	170.7	23.8	167.3	26.5	167.5	28.8	< 0.01
child cholesterol	168.4	26.9	166.7	25.8	167.5	28.9	167.6	27.0	168.0	27.9	0.90
adolescent cholesterol	162.3	27.8	157.3	25.4	161.0	28.1	162.5	27.2	160.7	28.3	0.02
adult cholesterol	182.1	31.5	184.4	35.7	177.8	32.5	176.6	34.4	179.2	35.9	< 0.01
systolic blood pressure	103.9	8.8	107.1	10.2	104.4	9.3	100.8	9.0	101.6	10.1	< 0.01
child SBP	97.3	8.0	97.7	8.9	97.9	8.6	96.9	8.6	96.8	8.8	0.19
adolescent SBP	107.6	8.2	107.6	7.7	107.8	8.9	106.8	8.2	107.5	8.7	0.11
adult SBP	110.1	9.8	111.6	10.5	110.7	10.5	109.0	9.8	110.2	9.8	< 0.01

*BMI* body mass index, *SBP* systolic blood pressure, *SD* standard deviation, *IQR* interquartile range

When the matched pregnancies were compared, agreement between the two sources was generally quite good, with kappa statistics > 0.9 for birthweight and 0.8 for gestational age (Table 2). Mean and median differences were close to 0. 128 births (7.5%) were reported as LBW and 1523 (88.8%) as not LBW by both sources; 47 (3.0%) were reported as LBW by the interview and not the birth certificate, while 18 (1.1%) were reported as LBW by the birth certificate but not the interview. 106 births (6.8%) were reported as PTB and 1340 (96.1%) as not PTB by both sources; 54 (3.9%) were reported as PTB by the interview and not the birth certificate, while 49 (3.2%) were reported as PTB by the birth certificate but not the interview.

**Table 2:** Comparison of reported birth outcomes vs. linked birth outcomes

	agreement (κ)^a^	mean difference^a^	CI	median difference
birthweight		−2.4	−17.8, 13.0	−0.1 g
low birthweight	0.95			
very low birthweight	0.98			
3-level low birthweight	0.94			
macrosomia	0.99			
gestational age		0.01	−0.09, 0.10	−0.50
preterm birth	0.81			
very preterm birth	0.87			
3-level preterm birth	0.79			
postterm	0.89			

^a^controlling for clustering within woman; medical records value – self-report

Few consistent predictors of discrepancy in reporting could be identified (Table 3, Additional file 1: Table S1). Black race was associated with an increased likelihood of discrepancy. First births had a higher likelihood of disparity in LBW and greater discrepancy in gestational age. Those with lower education were more likely to have a discrepancy in reporting LBW (though not birthweight) and in gestational age (though not PTB). Older age was generally associated with greater difference in gestational age, as was time since pregnancy. Cardiovascular risk factors did not show a consistent pattern of being associated with discrepancies in reporting, though occasionally there were statistically significant associations (childhood BMI and blood pressure for birthweight, adolescent cholesterol for PTB).

**Table 3:** Predictors of discrepancy in reporting birth outcomes, the Bogalusa Babies study

	discrepancy in LBW		discrepancy in PTB		difference in birthweight		difference in gestational age	
	OR	95% CI	OR	95% CI	beta	95% CI	beta	95% CI
Black race	2.77	1.67–4.61	0.97	0.72–1.31	−38	−70, −4.9	− 0.39	−0.58, − 0.2
ever smoker	0.86	0.50–1.48	0.82	0.58–1.16	−13	−47, 20	0.05	−0.16, 0.26
education								
< high school	3.71	1.58–8.74	0.65	0.33–1.27	16	−43, 75	−0.45	−0.84, − 0.05
high school	2.07	0.94–4.56	1.26	0.82–1.93	13	−32, 5	0.00	−0.28, 0.27
some college/associates	2.02	0.93–4.38	1.09	0.72–1.64	13	−24, 50	0.04	−0.20, 0.28
college+	1.00		1.00		0		0.00	
primiparous	1.72	1.06–2.80	1.01	0.75–1.34	18.6	−7.1, 44.3	−0.28	−0.48, − 0.08
age at youngest visit	0.98	0.92–1.05	0.98	0.93–1.02	0.52	−3.1, 4.1	0.03	0.00, 0.06
age at oldest visit	1.00	0.98–1.02	0.99	0.98–1.01	0.62	−0.47, 1.72	0.01	0.01, 0.02
age in 2018	0.99	0.95–1.02	0.98	0.96–1.00	1.41	−0.50, 3.33	0.03	0.02, 0.04
year at first visit	1.01	0.98–1.04	1.01	0.99–1.03	−1.48	−3.4, 0.4	−0.03	− 0.04, − 0.01
year at most recent visit	1.01	0.98–1.04	1.00	0.98–1.02	0.32	−1.43, 2.08	0.01	−0.00, 0.02
time since pregnancy	0.97	0.93–1.00	0.98	0.96–1.01	1.98	−0.46, 4.43	0.07	0.06, 0.09
year of pregnancy	1.04	1.00–1.08	1.02	0.99–1.04	−1.98	−4.43, 0.46	−0.07	− 0.09, 0.06
BMI	1.01	0.96–1.06	0.98	0.94–1.01	2.4	−0.14, 4.93	0.01	−0.01, 0.03
child BMI	1.01	0.93–1.10	0.98	0.93–1.03	6.6	2.61, 10.65	−0.02	− 0.05, 0.01
adolescent BMI	1.02	0.96–1.08	0.97	0.93–1.01	1.3	−2.34, 4.94	−0.03	−0.05, − 0.00
adult BMI	1.00	0.97–1.05	1.00	0.97–1.03	1.9	−0.59, 4.38	0.01	−0.01, 0.03
cholesterol	1.00	0.99–1.01	1.00	0.99–1.00	0.37	−0.19, 0.94	0.00	−0.00, 0.01
child cholesterol	1.00	0.99–1.01	1.00	0.99–1.00	−0.10	−0.75, 0.56	0.00	−0.00, 0.00
adolescent cholesterol	1.00	0.99–1.01	1.00	1.00–1.01	0.12	−0.56, 0.79	0.00	−0.00, 0.01
adult cholesterol	1.00	0.99–1.00	1.00	0.99–1.00	0.78	0.17, 1.39	0.00	−0.00, 0.01
systolic blood pressure	1.02	1.00–1.04	0.99	0.97–1.01	1.48	−0.39, 3.36	0.02	0.01, 0.03
child SBP	1.00	0.97–1.03	0.99	0.97–1.01	2.46	0.44, 4.48	0.01	−0.01, 0.02
adolescent SBP	1.02	0.99–1.05	1.00	0.98–1.02	1.21	−1.26, 3.69	0.01	−0.00, 0.03
adult SBP	1.01	0.98–1.04	0.99	0.97–1.01	0.81	−1.16, 3.24	0.02	0.00, 0.03

*LBW* low birthweight, *PTB* preterm birth, *BMI* body mass index, *SBP* systolic blood pressure

## Discussion

This analysis serves as background in assessing the likely degree of bias for the overall Bogalusa Babies study, which aims to determine the relationship between cardiovascular risk factors and pregnancy outcomes. Overall, there are two questions to be answered: when considering information about reproductive history in a long-term study with no original goal of assessing reproductive outcomes, does linkage to vital statistics or interview find more participants or more representative participants; and when both data sources are available, how do they compare? These questions are relevant not only to our own study, but to other studies who may be interested in studying the relationship of pregnancy outcomes with chronic disease, and those determining the best way to capture such information.

Generally, we found that consistent participation in the study was the best predictor of being located, via linkage, interview, or both. Black women were also more likely to be linked or interviewed, which differs from other analyses of loss to follow-up [28]. Previous studies of linkage to vital statistics indicate lower linkage of those living in deprived areas and rural areas [29], and that therefore, such studies may suffer from a bias in estimating social gradients of health. Studies also indicate increased attrition with lower SES [30]. To some extent, we found a small tendency for lower education to be associated with loss to follow-up, although in this case, those who seek higher education are likely to move from the area (a relatively small town with no university in the parish), at least temporarily. Other studies have also found that more frequent or more intense involvement in the study reduces attrition [31, 32]. Generally, clinical trials and longitudinal studies find those at increased medical risk, advanced-age, and young adult participants are more likely to drop out [30, 32, 33]. Smokers are also more likely to be lost to follow-up [28, 30, 33], which, again, was not the case in our study, although this is probably partly due to the fact that those lost at a young age might not have begun smoking at the time they participated in the study.

The major question of concern is whether use of one or both sources is likely to lead to biased estimation of the relationships. Overall, two-thirds of all participants were located by one or both sources. While 33% loss to follow-up is easily sufficient to bias an analysis, the sample size that remains is adequate for many research questions, so the concern is whether this sample is representative of the larger study. The analysis is generally reassuring on that point, as cardiovascular risk factors usually did not vary between those linked and those not, or those interviewed and those not. There was not a consistent profile indicating that those with worse or better health were systematically excluded, nor of exclusion of those with low or high socioeconomic status.

Agreement between sources for those included was generally quite good, although there was some indication that black race might have been associated with larger discrepancies in reporting, as well as time since the pregnancy. Several reasons for discrepancies can be imagined. They include 1. Poor memory; 2. Misassigning outcomes (i.e., mixing up birthweights of siblings); 3. Misunderstanding or lack of communication around medical issues (e.g., change in due date based on ultrasound not being communicated to or understood by a woman); 4. Approximation, particularly for full-term gestational ages and pregnancies occurring before the routine use of ultrasound; 5. Not regarding gestational age at birth as worth keeping track of, particularly for earlier births that were not ultrasound-dated and went to full term; 6. Data issues: incorrect linkage or data entry, although studies comparing medical records to vital statistics find that vital statistics data are accurate for birthweight and gestational age [34, 35]. Many of these factors are likely to be correlated with education and the effort and respect accorded a woman by medical providers, all of which are more likely to be provided to white women than black women. Black women also tended to have children earlier and thus had a longer time since pregnancy, although this did not fully explain the difference.

Overall, results are generally reassuring as to possible bias; the limited variation by cardiovascular predictors and the good quality of agreement about birth outcomes suggests that loss to follow-up or missed linkage is not likely to produce major bias for studies of those topics.

Our results are generally consistent with previous studies indicating that mothers remember the birthweight and gestational age of their infants quite well, even after many years [36, 37]. A few facts about self-report should be considered. In the U.S., women generally report birthweight in pounds and ounces, while vital statistics data are in grams; however, the conversion did not produce major issues. Perhaps more serious is that women often remember their babies’ gestational age in terms of weeks while medical records and vital statistics report in days; although we allowed for reporting in both weeks and days, most women reported only in weeks. We also began the interview asking whether the baby was early, late, or on time, and women generally reported the baby was on time if s/he was born within the week expected. The more precise recording in medical records and vital statistics is better for studies that treat gestational age as a continuous variable. Finally, many of the earlier births in this study occurred prior to routine ultrasound dating, so women may have had less exact dating available to them.

The question then arises as to whether these results apply to other studies. Some aspects of the study are unusual, though possibly relevant to other studies. Participants did not initially agree to be in a long-term study; particularly, the original waves of data collection were collected as cross-sectional studies rather than a planned longitudinal analysis. Therefore, the loss of participants who participated once, many years ago, as children, is not particularly surprising. This analysis also assesses only women, who are generally more likely to continue participation in studies [28, 38] but also more likely to change their last names. Any analysis addressing pregnancy will have this population. The geographic basis for the study also affects the follow-up; in this semirural area, higher-SES individuals are more likely to leave the area, which affects their loss to follow-up, not necessarily the case for more extensive studies or other types of areas.

## Conclusions

Combining information from multiple sources to increase sample size and outcome ascertainment may be valid. We have demonstrated support for use of data harmonization across sources as a feasible and valid way to create analytic epidemiologic cohorts. Studies will generally consider consistently-collected data such as vital records as the preferred source, but can be augmented with maternal self-report for these outcomes. This is good news for population health sciences’ ability to leverage the many existing, large-scale sources of data on health and health determinants for research that expands their scope further by answering previously unexplored questions, even those that the data were not initially collected to answer.

## Additional file

Additional file 1: **Table S1.** Predictors of discrepancy in reporting birth outcomes, the Bogalusa Babies study, multivariable analysis. (DOCX 20 kb)

## Data Availability

Data are available to qualified researchers upon request and completion of a data use agreement. Due to human subjects protections, data are not publicly available.

## Abbreviations

BHS: Bogalusa Heart Study
BMI: Body mass index
LBW: Low birthweight
PTB: Preterm birth
SES: Socioeconomic status
SSN: Social security number
