# Supplementary material for: Comparison of reproductive history gathered by interview and by vital records linkage after 40 years of follow-up: Bogalusa Babies
Source: BMC Med Res Methodol. 2019 Jun 4;19:114. doi: 10.1186/s12874-019-0758-0 (PMC6549375; doi:10.1186/s12874-019-0758-0)
Supplement: Supplementary file 1 — Table S1. Predictors of discrepancy in reporting birth outcomes, the Bogalusa Babies study, multivariable analysis. (DOCX 20 kb) [file 12874_2019_758_MOESM1_ESM.docx]

REQUESTED REVISIONS: 
"The variables assessed were largely those listed in Table 1." While I appreciate the response, the word "largely" does not answer the initial query. Unless the variables in table 1 are exactly those that were analyzed, then some explanation for why some variables might have been excluded is warranted.

**The “largely” in the response to the reviewer refers to the fact that some of the variables are functions of, or strongly correlated with, each other, so a single variable was chosen in a few cases, usually the one with most complete data or believed to be most relevant. Tested variables were:**

Race; education, 3 categories; ever smoked; age at youngest visit; age at oldest visit; age in 2018; year of first visit; year of last visit; time since pregnancy; mean BMI across all visits; mean childhood BMI; mean adolescent BMI; mean adult BMI; mean cholesterol across all visits; mean childhood cholesterol; mean adolescent cholesterol; mean adult cholesterol; mean systolic blood pressure across all visits; mean childhood systolic blood pressure; mean adolescent blood pressure; mean adult blood pressure; year of birth; education, 2 categories; self-reported financial status as a child; self-reported financial status as an adolescent; self-reported financial status as an adult.

After the initial reviewer query of whether one data source was consistently the source of the lower estimates, the authors' responses that "the differences were so small that they are not important" and the differences were "almost evenly split" are uncomfortably vague and non-quantitative. Let the data do the talking. Show us what the differences are, and let the reader decide if they were small, or whether the split was "almost" even. The imprecision of these responses does not fill me with confidence.

**This seems to be a response to our R to R the first time round. The data we summarized as so small they are not important were indeed included in table 2 (-2 g and 0.01 weeks, respectively). For the categorical variable (which one was higher?) that we summarized as almost evenly split, the proportions are birthweight, 45% vs. 55%; gestational age, 51% vs. 49%.**
